# A powered prosthetic ankle joint for walking and running

**DOI:** 10.1186/s12938-016-0286-7

**Published:** 2016-12-19

**Authors:** Martin Grimmer, Matthew Holgate, Robert Holgate, Alexander Boehler, Jeffrey Ward, Kevin Hollander, Thomas Sugar, André Seyfarth

**Affiliations:** 1 Lauflabor Locomotion Laboratory, Institute of Sports Science, TU Darmstadt, Magdalenenstraße 27, 64289 Darmstadt, Germany; 2 Springactive, Inc., 2414 W. 12th Street Suite 4, Tempe, AZ 85281 USA

**Keywords:** Prosthesis, Walking, Running, Ankle, Power, Work, Joint

## Abstract

**Background:**

Current prosthetic ankle joints are designed either for walking or for running. In order to mimic the capabilities of an able-bodied, a powered prosthetic ankle for walking and running was designed. A powered system has the potential to reduce the limitations in range of motion and positive work output of passive walking and running feet.

**Methods:**

To perform the experiments a controller capable of transitions between standing, walking, and running with speed adaptations was developed. In the first case study the system was mounted on an ankle bypass in parallel with the foot of a non-amputee subject. By this method the functionality of hardware and controller was proven.

**Results:**

The Walk-Run ankle was capable of mimicking desired torque and angle trajectories in walking and running up to 2.6 m/s. At 4 m/s running, ankle angle could be matched while ankle torque could not. Limited ankle output power resulting from a suboptimal spring stiffness value was identified as a main reason.

**Conclusions:**

Further studies have to show to what extent the findings can be transferred to amputees.

## Background

The current standard for prosthetic ankle joints are passive SACH (solid ankle cushioned heel) or carbon fiber ESAR (energy storage and return) feet. In contrast to the stiff SACH feet, ESAR feet are able to store energy during the stance phase and release it later during push-off [1, 2]. Through this they are able to mimic the function of the Achilles tendon [3]. In contrast to human muscles, carbon feet are not able to create net positive work for ankle plantar- or dorsiflexion. Thus, actuation systems are required to achieve able-bodied ankle behavior. Different approaches with pneumatics [4] and electric motors [5–11] have been developed in recent years. In order to support amputees in common daily life activities like walking on flat terrain [10], stairs [12] or slopes [13], these activities were investigated and biomechanical characteristics were implemented in the active ankle joints.

In addition to the daily life movement requirements, amputees want to participate in social activities like sports. Cycling, swimming, and running are some possible sporting activities, with running also being fundamental to multiple activities such as ball games. Some special prosthetic solutions for different sports have been designed [14–16] such as waterproof legs and arms for swimming, electronic controlled knee joints with programmable modes specifically for cycling or skiing, (C-Leg, Otto Bock) as well as running and sprinting prostheses. As the limited range of motion (ROM) of existing prosthetic walking feet used for daily life make it unfavorable to run, amputees typically change their prosthesis when running. Ankle prosthesis designs for running and especially for sprinting have no heel element to make it possible to roll over the foot, resulting in a gait similar to forefoot running. The missing heel element increases effort for standing and other tasks of daily life and therefore makes the feet designed for running less appropriate for daily usage. Nevertheless, the limitations in ROM [17] passive walking feet can be also used for running. Czerniecki et al. [2] compared different generations of passive feet at 2.8 m/s transtibial amputee running. They found that a passive SACH (solid ankle cushioned heel) foot was only able to return 31% of the stored energy. ESAR (energy storage and return) feet were able to return 52% (Seattle Foot) to 84% (Flex Foot) of energy. For the Flex Foot about 0.19 J/(kg m) returned (stride length 2 m). In comparison, about 0.35 J/(kg m) of positive work at the ankle joint are required to perform running for able-bodied at the same speed [18]. Positive work output for different running feet (Flex Run, Cheetah, Catapult) was about 0.1 J/(kg m) when evaluating various running speeds (2.5–3.5 m/s, [19]). Prosthetic foot peak power output of the same study was much lower (2.2–2.9 W/kg) compared to able-bodied running data (8.7 W/kg, 2.6 m/s, [18]).

A powered ankle prosthesis can provide positive work and overcome the limitations of ROM. Therefor sufficient motor power especially for higher walking and running speeds is required [20]. Calculations show that different arrangements of elastic elements can decrease these requirements, especially in running gait [21, 22]. When considering the assistive effect of a series spring to mimic the ankle joint torque-angle curves with a motor, about 0.6 to 1.3 W/kg mechanical peak power output should be provided in walking (1.1–1.6 m/s) and 2.6–2.8 W/kg [20] for medium marathon running speeds of 2.6–3 m/s [23]. A running speed of 4 m/s would require four times (3.9 W/kg) the motor peak power of the preferred walking speed.

Along with reduced power demands, elastic elements can also reduce energy requirements for a powered prosthesis. Modeling of a powered ankle prosthesis (to match able-bodied torque-angle profile) demonstrated that walking would require about 0.14 J/(kg m) to 0.18 J/(kg m) (1.1–1.6 m/s) and running about 0.22 J/(kg m) (2.6, 3 and 4m/s) mechanical work input when using a series spring for assistance [20]. The design of a powered running prosthesis should be adapted to meet the increase of about 22 to 57%.

In addition to the power and energetic requirements, the control needs an adaptation to distinguish between walking and running and the desired speed. This could be critical when amputees want to run below or walk above preferred transition speed (2.1 m/s, PTS, [24]). Transitions from walking to running and running to walking must be realized quickly to support acceleration or deceleration during the transition process. Transition between movement and standing are critical for safety.

To investigate these topics, powered prosthetic ankles were designed to be capable of walking and medium speed running. First concepts were published by Bellman et al. in [25]. The proposed design included two motors to realize tasks like jumping and running. Based on this idea a first running ankle was built in 2009 and tested with a military amputee [26, 27]. Evaluations demonstrated that the weight of the system and inertial properties of the actuators had to be improved in follow up versions.

The next generation of a powered running ankle was built in 2012. Instead of two brushed 150 W motors a brushless 200 W DC motor was used for the Walk-Run ankle. A controller to change between standing, walking, and running with speed adaptation was developed. For a first proof of concept, the system was evaluated with one able-bodied subject wearing the prosthesis in parallel to the fixed healthy foot. This article includes the results of the biomechanical evaluation of this prototype and the comparison to able-bodied subject data and corresponding prosthetic model estimations. Next to powered ankles also combined system with powered knee and ankle have been evaluated while running [28]. Control concepts were developed to perform walk-run transitions [29].

The evaluation of the control response to speed changes and gait transitions is not a part of this study. The topic should be addressed in future work.

## Methods

### Design of the Walk-Run ankle 

**Fig. 1:**
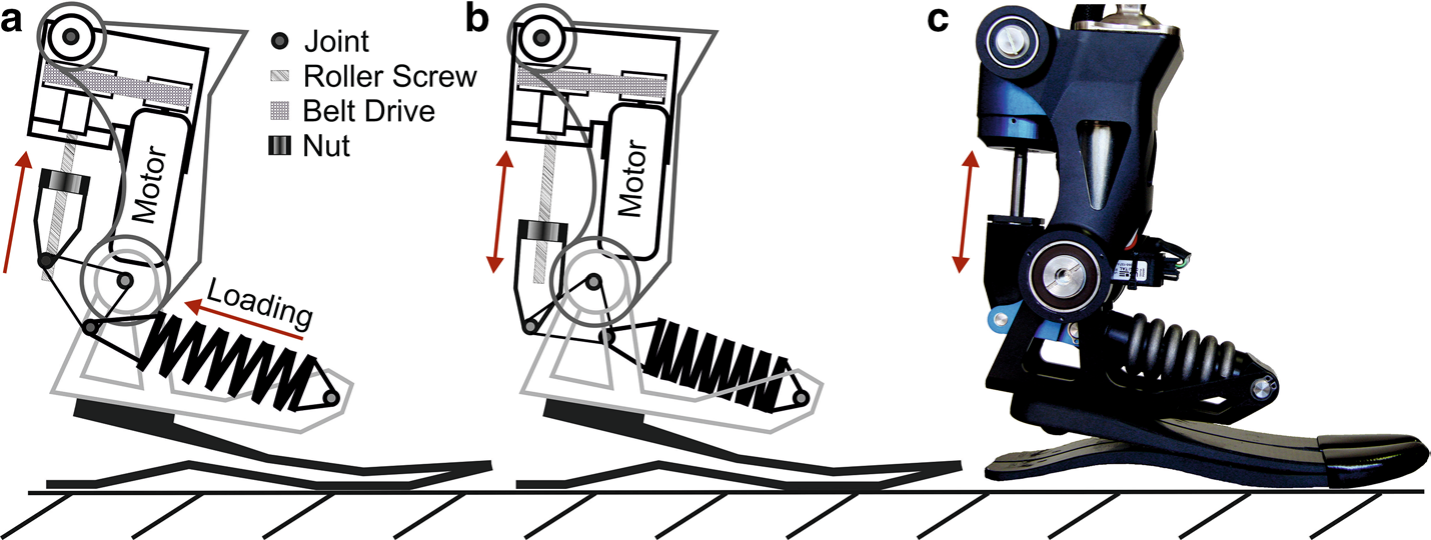
Walk-Run ankle (Springactive): the motor powered prosthesis to investigate on walking and running is shown under load (**a**) and without spring deflection (**b**,** c**). The prosthesis consists of 4 major parts. The carbon foot, the foot adapter (*light gray*), the main housing (*gray*), and the motor gear mounting (*black*). The foot adapter includes the anterior spring attachment. The main housing has the ankle joint at the distal end and the connection to the motor and gear mounting at the posterior proximal end. A small triangular linkage connects the spring with the nut. This linkage is guided by a third attachment point that is in line (but no rigid connection) with the ankle joint. When the roller screw rotates the nut will move up or down to cause a joint torque at the ankle joint. In flight phase the movement of the nut will directly cause ankle plantarflexion or dorsiflexion. During stance phase the nut direction defines if the spring will be loaded or unloaded to modulate desired ankle tourque-angle profiles

The Walk-Run ankle (Fig. 1) is an active ankle prosthesis that was designed in 2012 to perform walking and running in lab conditions. It is the first generation of a series of improved powered ankles for substantial load demands. The system uses a 200 W brushless DC motor as a power source and a spring to benefit from elastic energy storage and release during walking and running gaits. The stiffness of the spring (445 kN/m) is optimized to reduce peak power and energy requirements through a modeling approach for a subject weight of 61 kg in a medium running speed of 2.6 m/s. The stiffness of the foot is not included in the model. To minimize deformation of the additional series elastic carbon foot, the stiffest possible version of the Pacifica LP foot (Freedom Innovations) was selected.

The current design has a weight of 1.9 kg, not including the battery and the electronics. Both the PC-104 used for control and the 4400 mAh 25.9 V battery (powering ankle and PC-104) are stationary on a test bench. For a subject (61 kg, 1.7 m height) with an amputation height of 0.31 m, the amputated limb mass is estimated to be about 2.3 kg according to calculations using the equation from [18]. Thus, the weight of the artificial foot, including the adapters and socket, is only slightly higher compared to the healthy condition. A belt drive is used as a transmission between the motor and roller-screw.

### Control of the Walk-Run ankle

**Fig. 2:**
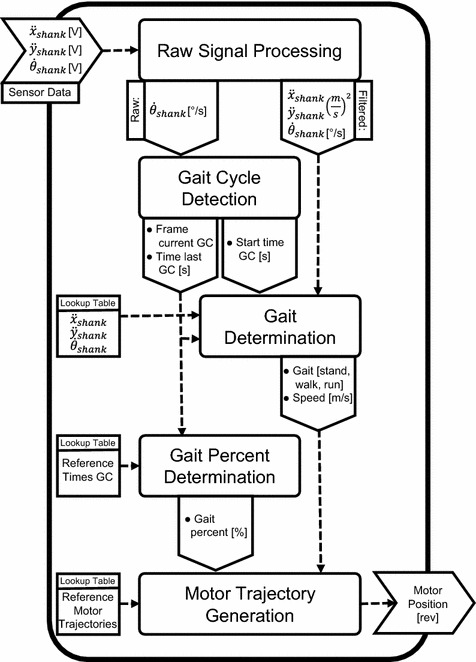
Controller flowchart: the sensor input data for the control is shank angular velocity $\dot{\theta }_{shank}$ and shank acceleration $\ddot{x}_{shank}$ and $\ddot{y}_{shank}$. Five main steps of data processing are required to define the motor trajectory output signal for the Walk-Run ankle. The Raw Signal Processing, the Gait Cycle Detection, the Gait Determination, the Gait Percent Determination, and the Motor Trajectory Generation. Three lookup tables from experimental gait studies of able-bodied subjects are required to run the control. One is including data similar to the sensor input as reference for determining the gait, one is including reference stride times to determine gait percent, and one is including the reference of the speed and gait dependent motor trajectories calculated in advance using the methods published in [31, 32]. If intermediate speeds are performed trajectories are interpolated

A controller for standing, walking, running, and transitions was designed using Simulink (MathWorks) to test the prosthetic prototype (Fig. 2). Two sensors, fixed at the upper part of the prototype, are implemented for gait control. A rate gyro sensor ($\dot{\theta }_{shank}$) to measure shank velocity is combined with a two axis accelerometer. For the accelerometer, $\ddot{x}_{shank}$ is oriented forward and $\ddot{y}_{shank}$ is oriented vertically when the subject is standing. All sensors measure motion in the sagittal plane.

All three signals (1000 Hz) were converted from voltage to acceleration or velocity in the “Raw Signal Processing” block. In addition, all the data was filtered for “Gait Determination” with a 2nd order Butterworth filter using a cutoff frequency of four to get a general representation of the gait behavior.

**Fig. 3:**
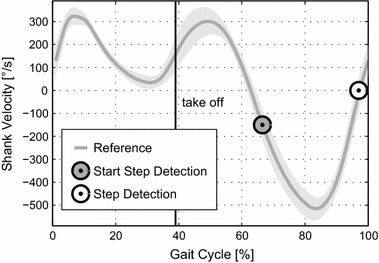
Step detection: human reference shank velocity for 2.6 m/s running used for stride detection (mean of 21 able-bodied subjects). Zero crossing from negative to positive is used to determine the beginning of the next stride. Step detection starts at −150 °/s to prevent from errors that could occur by zero crossings at lower speeds during stance

Three parameters must be determined in order to determine the appropriate motor position: the current gait mode, the gait speed, and the gait progression percentage. To determine these parameters, first a “Gait Cycle Detection” is done. The beginning of the gait cycle is identified by rate gyro sensor data from the shank. When shank angular velocity crosses from negative (swinging forward) to positive velocity (swinging backward) the beginning of the gait cycle is defined (Fig. 3). At some walking and running speeds the same zero crossing can occur during Midstance. To avoid errors in stride detection a second condition was defined. During the swing phase, angular velocity reaches a maximum. For slower speeds the maximum value decreases. At 0.5 m/s walking speed the maximum was about −200 °/s. A threshold condition of −150 °/s needs to be fulfilled before shank velocity zero crossing can be used to identify the beginning of a stride. The output of the “Gait Cycle Detection” is the current frame and subsequently the start time of the gait cycle. In combination with the start time of the last gait cycle the time of the last stride can be determined.

In the “Gait Determination” the incoming three sensor signals are compared to sensor reference trajectories starting at the beginning of gait cycle. The most similar reference is identified calculating a cumulative sum of error between the curves. This results in a measurement of the current gait mode and the movement speed.

The “Gait Percent Detection” block uses a lookup table of times, $t_{cyc}$ (s), of gait cycles from different speeds of walking and running [24]. Knowing gait mode and speed, the appropriate value can be identified and used to calculate gait percent $G_\%$ in combination with the current frame value $G_fr$ (1000 Hz).

1 $$\begin{aligned} G_\% = (G_{fr} / t_{cyc}) / 10 \end{aligned}$$

Due to individual differences in $t_{cyc}$ from the reference lookup table, $t_{cyc}$ of the last stride is also taken into account as long as gait mode and speed are not changed. This results in more precise gait percentage detection.

As a last step, the gait mode, the speed, and the gait percentage are used in the “Motor Trajectory Generation” block to determine the motor trajectory using a reference lookup table.

The desired motor trajectory is followed by the implementation of PD control. PD values enabled the system to follow the desired motor trajectories with a mean difference of 0.2 mm over the gait cycle in 1.6 m/s walking. As previously described, a belt drive is used in conjunction with a roller-screw to effect dorsiflexion or plantarflexion at the prosthetic ankle joint.

### Control and model reference data

Three different experiments were used to define reference data for the prosthetic control.

In the first experiment [24], able-bodied (21 subjects) walked and ran (0.5–2.6 m/s) on a treadmill with integrated 3D force sensors (Kistler, 1000 Hz). Kinematics were recorded by high-speed infrared cameras (Qualisys, 240 Hz). A second, similar experiment (7 subjects), was repeated for higher running speeds (3–4 m/s). Ankle angles and torques were calculated [24, 30] and used to determine motor trajectories for the powered ankle based on the method presented in [31, 32]. The used method determines the deflection of the Walk-Run ankle spring when applying recorded torque data from the able-bodied experiments. Motor trajectories must accomplish the length change to match human ankle angle data.

Reference times, used in the controller to determine the gait percent, were taken from both treadmill experiments.

In the third experiment, one subject (height: 173 m, mass: 63.5 kg, age: 23, male) walked and ran with the same speeds as in the previous experiments over level ground. The pace was set by a bicycle. A two axis accelerometer and a rate gyro sensor were mounted to a small wireless board. The board was affixed at the shank near the ankle joint to measure reference values for all gait conditions. The fixation height of the system was similar to the height of the sensors of the Walk-Run ankle. About 40 strides were measured for each condition at the instrumented leg. Mean values for the gait cycle were created and filtered in like manner to the incoming data in the prosthetic controller (2nd order Butterworth filter, cutoff frequency 4 Hz). The frequency was selected in order to get the general shape of the signal rather than measuring the real kinematics.

All the experiments were in compliance with the Helsinki Declaration.

### Evaluation of Walk-Run ankle

**Fig. 4:**
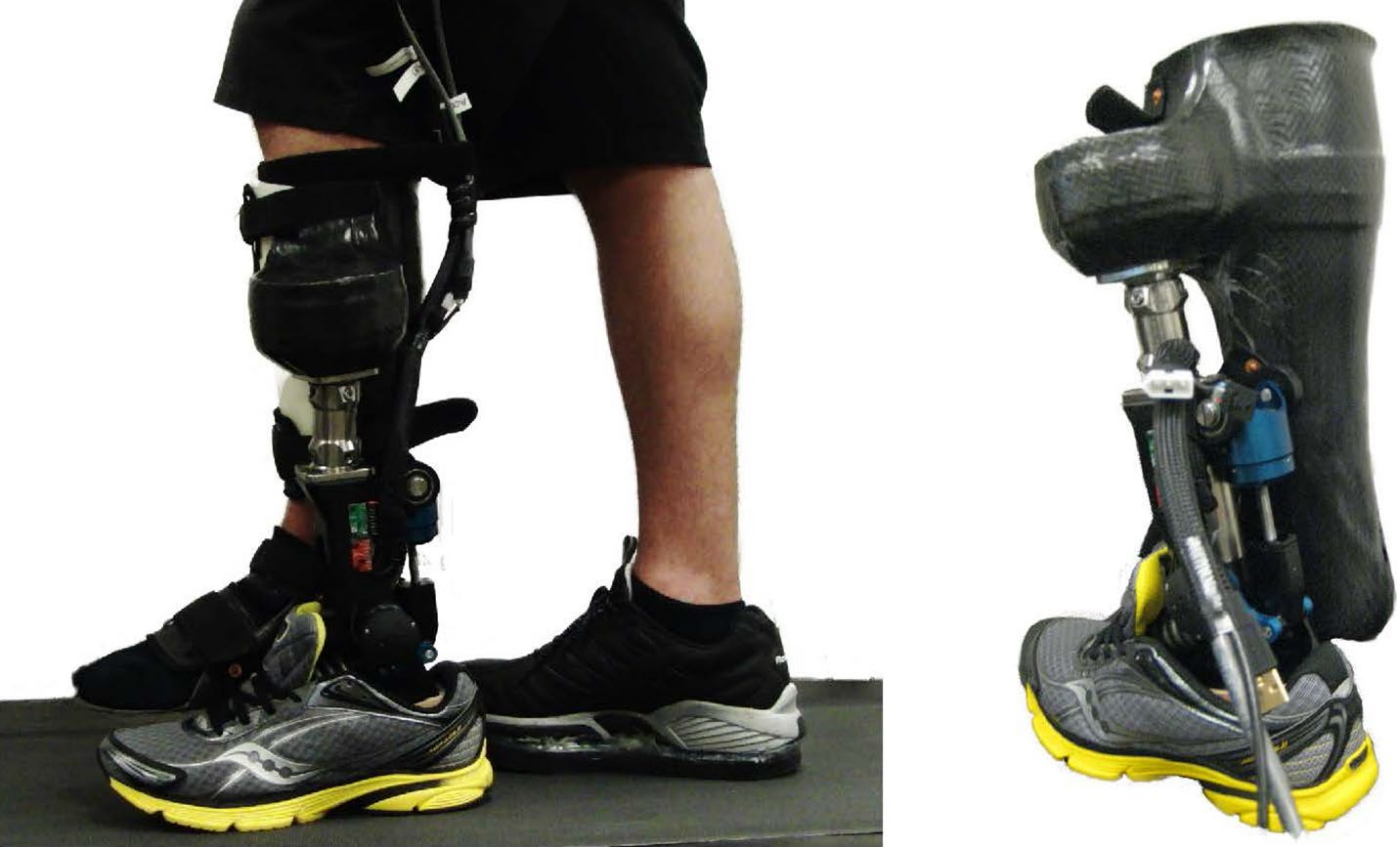
Bypass test orthosis: the Walk-Run ankle was mounted in parallel at an orthotic bypass to test it at an able-bodied subject

For the first evaluation of the Walk-Run ankle, one subject (similar to third preparation experiment) was tested on a treadmill using a bypass system (Fig. 4). The prosthesis is linked in parallel by an orthosis to a healthy subject. The leg length on the prosthetic side increased by about 5 cm. The shoe of the opposite leg was equipped with an additional 1.5 cm sole to reduce differences in leg length. Various walking and running speeds were tested. For this evaluation, 44 continuous strides of 1.6 m/s walking, 60 continuous strides of 2.6 m/s running, and 37 continuous strides of 4.0 m/s running were used.

### Calculation of biomechanical parameters

Biomechanical parameters of the Walk-Run ankle were calculated using the motor encoder and the ankle angle encoder. The length of the spring was determined by the position of the nut (motor) in combination with the ankle angle. Using Hooke’s law, the spring force could be calculated. Spring and nut velocity were determined by numerical differentiation. By using the lever arm of the spring towards the ankle joint, ankle torque was calculated. Ankle torque could be related to force applied by the nut using the lever arm of the ankle joint towards the roller screw. By multiplying spring velocity and spring force the mechanical power was calculated. Nut output power was calculated by multiplying nut force and nut velocity. The sum of spring power and nut power is equal to the ankle joint power.

Stride length of the bypass site was calculated using the speed of the treadmill and the time required for the gait cycle.

## Results

### Gait quality

#### Walking 1.6 m/s

**Fig. 5:**
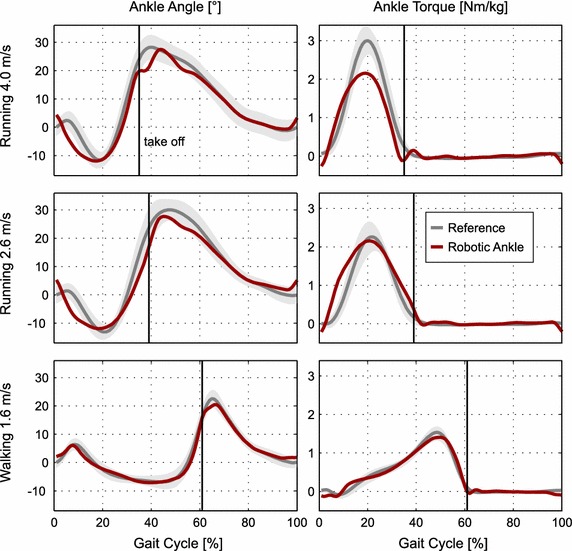
Angle and torque: ankle angle (*left*) and ankle torque (*right*) for the mean of multiple strides using the Walk-Run ankle (*red*) and the mean of up to 21 able-bodied reference subjects (*gray*). For the reference data also standard deviation is shown. Data is presented for the gait cycle of 1.6 m/s walking, 2.6 and 4.0 m/s running. The take off of the reference is indicated by a* vertical line*. An increase of the ankle angle implies plantarflexion

**Fig. 6:**
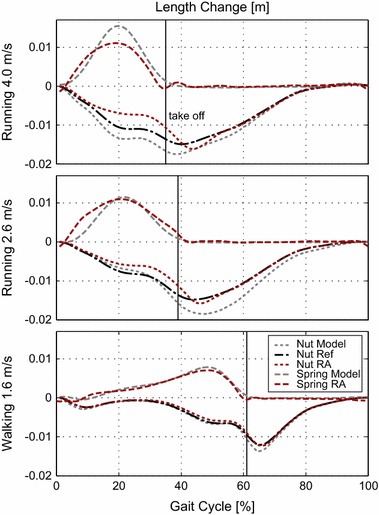
Nut and spring length change:length change of the nut (*dotted*) and the spring (*dashed*) for the mean of multiple strides using the Walk-Run ankle (*red*) and the mean of up to 21 able-bodied reference subjects (*gray*). Data is presented for the gait cycle of 1.6 m/s walking, 2.6 and 4.0 m/s running. The take off is indicated by a* vertical line*. Positive values imply lengthening.* Light gray color* indicates the pattern for the theoretical model. As detected locomotion speed is different from the desired model and the treadmill speed real nut reference (*black*) curves differ from the model trajectory

**Fig. 7:**
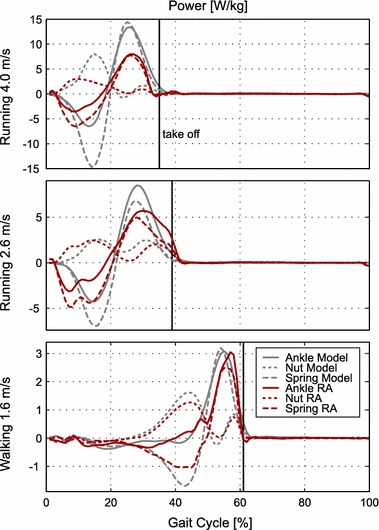
Nut, spring and ankle power: mechanical power of the nut (*dotted*), the spring (*dashed*) and the ankle (*solid*) for the mean of multiple strides using the Walk-Run ankle (*red*) and the mean of up to 21 able-bodied reference subjects (*gray*). Data is presented for the gait cycle of 1.6 m/s walking, 2.6 and 4.0 m/s running. The take off is indicated by a* vertical line*. As detected locomotion speed for running was different from the theoretical model and the treadmill speed, differences in power curves are to some extend caused by changes in the interpolated nut trajectories

The ankle angle sensor output and the calculated torque of the Walk-Run ankle were compared to healthy subject data. We found that for walking at 1.6 m/s the controller identified a mean speed of 1.6 m/s during the gait cycle. Stride length (1.72 m) was a little higher compared to the mean of the reference subjects (1.51 m). The angle and the torque matched almost perfectly (Fig. 7) to reference data. Power curves and related energy of the model calculations [0.17 J/(kg m)] differ to some extent from the values measured by the Walk-Run ankle [0.14 J/(kg m)]. During the loading phase we could identify small differences between the desired nut reference and the robotic nut trajectory (Fig. 6). As a result, the peak power exerted by the motor to load the spring is smaller than in the model (Fig. 5 at 45%). As a consequence, the energy saved and released by the spring [0.11 J/(kg m)] is also less compared to the model [0.15 J/(kg m), Table  1]. A small power peak was identified at about 50% of the gait cycle. The peak is caused by a small delay in elastic energy return of the spring. As opposite leg touch down typically occurs at the same time it might be a consequence related to double support.

**Table 1:** Mechanical work provided by the motor (nut output, positive + absolute of negative), the spring (positive) and the overall robotic ankle in J/(kg m)

	Nut		Spring		Robotic ankle	
Trial (m/s)	RA	Model	RA	Model	RA (tot/pos/neg)	Model (tot/pos/neg)
Running 4.0	0.12	0.22	0.17	0.31	0.29/0.2/0.09	0.49/0.35/0.14
Running 2.6	0.21	0.23	0.23	0.25	0.44/0.32/0.12	0.51/0.36/0.15
Walking 1.6	0.14	0.17	0.11	0.15	0.2/0.15/0.05	0.24/0.19/0.05

Model assumptions were made using the stiffness of the spring and the data from [24]. Lower model requirements are possible if the spring stiffness is optimal for each individual gait condition and the individual subject weight

The maximum power output of 3 W/kg is almost equal, but the timing of the maximum power of the Walk-Run ankle is later compared to the model curve.

#### Running 2.6 m/s

While running at a treadmill speed of 2.6 m/s, the Walk-Run ankle identified a mean speed of about 2.8 m/s when using the bypass. The stride length of the subject was 1.92 m compared to 1.94 m for the reference data. When comparing the ankle angle and the torque to reference data from 2.6 m/s, especially at touch down and take off, differences could be identified. The ankle angle begins dorsiflexion earlier (0–10%) and plantarflexion later (30–50%) compared to reference running data (Fig. 7). The difference in shape of the torque is in line with the observed changes in ankle angle. The nut did not follow the reference trajectory closely during the stance phase (Fig. 6). The length of the spring increases earlier and decreases later compared to reference data. Power curves differ in shape from the model data. The amount of work provided by the robotic ankle motor [0.21 J/(kg m)] and by the spring [0.23 J/(kg m)] is similar but not exactly that of the model [0.22 nut output and 0.25 J/(kg m) spring, Table 1]. Maximum power output is about 5.7 W/kg combining the effect of the spring and the motor. This maximum power output is much lower than the 8.5 W/kg of the model.

#### Running 4.0 m/s

For running at a treadmill speed of 4.0 m/s, the controller identified a speed of 3.7 m/s. The reference group used a stride length of 2.81 m at 4 m/s running. The subject with the bypass had a mean stride length of about 2.67 m. When comparing to the reference data of 4.0 m/s, we identified the same ankle angle behavior as in 2.6 m/s running. Dorsiflexion happens earlier and plantarflexion later compared to the model. After takeoff, a larger difference between model and system ankle angle occurs. Similar fluctuations after takeoff with less amplitude can be found for 2.6 m/s running and 1.6 m/s walking. Similar to running at 2.6 m/s, there is a gap between the reference nut position and the real nut position in the stance phase. The spring is not elongated in the same way as the model (Fig. 6). As a result of nut position and spring length, ankle torque is less compared to the model values (Fig. 7). In addition, peak power and work [nut output: 0.12, spring: 0.17 J/(kg m)] are less compared to the model data [nut output: 0.22, spring: 0.31 J/(kg m), Table  1]. Joint peak power should be about 13.4 W/kg. The robotic ankle can provide about 8 W/kg in total combining the effect of the spring and the motor.

## Discussion

### Replication of human gait

The Walk-Run ankle could mimic reference subject walking and running behavior when testing using a bypass system on a healthy subject. When comparing the reference and the experimental data only subtle differences could be identified in walking. For running at 2.6 m/s minor differences for the ankle torque and the ankle angle exist. Work output at the ankle at this speed is comparable to able-bodied data. Thus we demonstrated that the Walk-Run ankle can overcome limitations in ROM [17] and in generating positive work compared to passive prosthetic walking (used for running, [2]) and running [19] feet. Human like ROM was also achieved at 2.25 m/s running for the Vanderbilt prototype [28] and at 2.5 m/s running for the Ruggedized Odyssey Ankle [33] during amputee testing.

At 4.0 m/s the biomechanics of the Walk-Run ankle differ from able-bodied gait. The positive work output is reduced compared to the model reference but still double compared to passive running feet (model 0.35, RA 0.2, passive 0.1 J/(kg m), [19]). To some extent differences between model and experiment could be caused by the difference of the detected speed (3.7 instead of 4.0 m/s) and the resulting difference in desired motor trajectory. In running, a gap between the desired nut reference (Nut Ref) and the achieved nut position of the robotic ankle exists (Nut RA) between 10 and 43% of the gait cycle.

As motor current is not at its maximum (almost reached between 16 and 28%) during some part of this period, it could be possible to reduce the difference by an adaptation of the control parameters. During Midstance (4 m/s), the motor was not able to deliver enough torque to move the nut, but motor torque was enough to hold position. It could be tested to what extent an earlier pretensioning of the motor, when less torque is applied, could improve elastic recoil.

The differences in nut trajectory during stance cannot explain the difference in ankle angle mainly caused by the spring deflection at the beginning of the stance phase. The spring is elongated faster than the model predicts. For a comparable joint torque, such a behavior could appear when spring stiffness is too low. Additional reasons for the increase in ankle torque could be a subject specific gait pattern, differences of the prosthesis to the human in structure and control, but also asymmetries caused by the bypass system. Shank velocity data and video show that the subject is touching the ground while the shank is in forward rotation. This is in contrast to the natural pattern where at touch down the shank is already in backward motion. The so called leg retraction [34] is used to avoid impacts. The longer leg (about 3.5 cm) of the bypass system seems to cause an increased impact that results in higher ankle torques compared to the reference data. For a more detailed analysis on the cause, motion capturing and force measurements of both legs would be required.

### Model assumptions on the energy flow

The modeling approach to determine the optimal energetic interaction of the spring and the motor uses mean values of torque and angle data of several subjects as an input. Based on the torque related spring deflection it calculates the corresponding length change for the nut to mimic desired ankle angle. It is assumed that lower extremity kinematics and kinetics will be similar when using the Walk-Run ankle including nut trajectories based on this approach. The data shows that when using the bypass design such an assumption seems to be acceptable for walking. Higher model agreement of the energy flow between the nut output and the spring was achieved without the large differences in leg length and leg mass when testing the Walk-Run ankle at an unilateral amputee [35].

In contrast, the running power curves show (Fig. 5) large differences between model predictions and achieved robotic ankle performance. Due to the results for amputee walking, the authors assume that the majority is caused by the bypass testing approach (leg length, mass) and the Walk-Run ankle limitations in peak torque. In addition some part of the differences may result from individual gait characteristics but also structural differences of the device compared to biology. Several muscles are represented by just one actuator and a biarticular coupling to the thigh is missing.

The powered ankle was designed to add additional functionality, to reduce user effort during locomotion, and to regain interlimb symmetry (unilateral amputee). Also when the energy flow between the spring and the motor is comparable to the model assumption and the torque and angle curves are almost similar to the reference, a transtibial amputee must not necessarily have lower locomotion effort combined with higher symmetry. Thus, further measurements are required to evaluate the user benefits of having powered assistance during running but also walking at the ankle joint. In level walking benefits for preferred walking speed [10], oxygen consumption [36], and reductions in stance to swing duration asymmetries [10] could be demonstrated.

### Spring fluctuations after take off

After takeoff, fluctuations of the spring occurred. This was more distinctive for the higher speeds. It is unclear how much these fluctuations in the early flight phase affect the gait stability. It could be worthwhile to investigate if it is possible to reduce fluctuations by using the motor for damping. As a result of the fluctuations, ankle angle after takeoff differs from reference data especially at 4.0 m/s running.

### Touch down detection

For ankle torque and ankle angle in running, small changes happen slightly before the detected start of the gait cycle. This could indicate that the gait cycle is not detected correctly. Shank velocity is used as an indicator for detecting the beginning of each new stride. For the reference data, zero crossing of the shank velocity data happens 3% before touch down (running 2.6 m/s, Fig. 3). As a result, the beginning of the gait cycle should be detected slightly before ground contact. In the experimental data, the opposite seems to happen. Ankle torque can be calculated before a new gait cycle is detected. The change in ankle angle shortly before the detected touch down is about 3° for both running speeds. As nut position is almost constant in this phase, the spring deflection (determined by the change of the ankle angle encoder) causes the calculated torque based on Hooke’s law. The change for the ankle angle encoder might be due to play in the mechanical system. On the other hand it could indicate that the subject touched the ground about 3% before the detected start of the gait cycle. As already discussed in subsection , shank velocity and video indicate that the bypass touches the ground while the shank is still in forward rotation. Ground reaction forces would be required to clarify the touch down timing. If the detection was out of time, the gyro control approach could be improved for higher accuracy. Calculated forces from the spring deflection could be used as an indicator for the beginning of a gait cycle. In addition to the method of using only gyro data, stance and swing phase can potentially be identified (Fig. 7). Future tests that include the bypass test bed should have a similar leg length to reduce the observed detection problems.

### Limitations of the Walk-Run ankle

The Walk-Run ankle nut was able to deliver about 3 W/kg mechanical peak power at 4 m/s running for a subject weight including bypass and prosthesis of about 67 kg. This 200 W maximum was almost reached for 2.6 m/s running. For running at 4.0 m/s, the model predicted a required nut peak power output of 8 W/kg for the chosen stiffness. The motor was not able to deliver these 536 W. Using an optimal stiffness could reduce requirements for running at 4 m/s to 3.9 W/kg [20]. This could greatly improve performance for the fastest running speed.

Peak torque limits during the experiment were at about 140 Nm. This limit was reached in both running conditions while in 4 m/s running it clearly limited the powered ankle performance.

Also, while the desired and achieved motor trajectories for 4 m/s running were not matching, the final ankle angle was almost equal (neglecting angle caused by fluctuations after take off) to the reference data. Thus we assume there could be a better solution that is a compromise between following of the desired motor trajectory and resulting similarity to able-bodied gait biomechanics and symmetry measures. Precise following will require a large amount of energy but gait quality may only improve slightly. Investigations on this topic could be made by measuring kinematics and kinetics to compare symmetry, joint work, limb loading, locomotion speed, balance, and by using a spiroergometry system to measure user effort.

## Conclusions

For the first preliminary study on the Walk-Run ankle, we found that it is possible to mimic human reference ankle joint behavior for walking and running up to a speed of 2.6 m/s. It was demonstrated that the Walk-Run ankle can overcome limitations in ROM and in generating positive work compared to passive prosthetic walking and running feet.

Compared to the reference data, at 4 m/s running, ankle torque from the prosthesis was not adequate. As the desired ankle angle was almost achieved even without the correct torque, the authors think that it is worthwhile to investigate the compromise between following calculated motor trajectories and achieved similarity to able-bodied gait biomechanics. The authors believe that there is a high potential to decrease peak power and energy requirements when motor trajectories are manipulated in a way to have reduced acceleration and less changes in direction.

Some hardware and control issues, like differences in motor desired and achieved trajectory, fluctuations of the spring, and touch down detection, could be identified. Some of these may occur due to the bypass setup. Thus, the next step would be to test the ankle on an amputee to avoid larger differences in leg length or mass distribution in between legs.

## Outlook

The data collected and presented in the paper was derived from the controller M. Grimmer developed for this system. This information is being used to influence the design of a ruggedized powered ankle that also has running capability. The new and improved design is being updated by SpringActive to include weight reducing titanium springs that have a longer life, higher efficiency bearings that increased system efficiency, and embedded electronics. First preliminary data can be found in [33].
